# Poor Prognosis and Therapeutic Responses in LILRB1-Expressing M2 Macrophages-Enriched Gastric Cancer Patients

**DOI:** 10.3389/fonc.2021.668707

**Published:** 2021-08-09

**Authors:** Yawei Zhang, Han Wang, Xiaoyu Xu, Huifang Liu, Tengfei Hao, Songcheng Yin, Changhua Zhang, Yulong He

**Affiliations:** ^1^Department of Center for Digestive Disease, The Seventh Affiliated Hospital of Sun Yat-sen University, Shenzhen, China; ^2^Department of Gastrointestinal Surgery, The First Affiliated Hospital of Sun Yat-sen University, Guangzhou, China; ^3^Department of Gynecology and Obstetrics, The Seventh Affiliated Hospital of Sun Yat-sen University, Shenzhen, China

**Keywords:** gastric cancer, LILRB1, tumor-associated macrophages, prognosis, tumor immune microenvironment

## Abstract

Immunosuppressive molecules are valuable prognostic biomarkers across different cancer types. Leukocyte immunoglobulin like receptor subfamily B1 (LILRB1) is considered to be an immunosuppressive molecule, which is an important receptor of human leukocyte antigen G. However, the clinical significance of LILRB1 expression in gastric cancer remains unexplored. We analyzed the immunohistochemistry data of 166 gastric cancer patients to determine the clinicopathologic and survival significance of LILRB1. Immunofluorescence was conducted to detect the co-localization of LILRB1 with infiltrating immune cells. Additionally, we also assessed the immune contexture, immune cell functions and tumor microenvironment state related to LILRB1. We found that LILRB1 was mainly present in tumor stroma which was higher in tumor tissues compared with matched adjacent tissues. High-LILRB1 expression was associated with more advanced tumor stage, higher recurrence risk and worse survival. Immunohistochemistry and bioinformatic analysis showed that LILRB1 had a significant positive correlation with M2 tumor-associated macrophages (TAMs) infiltration. Immunofluorescence confirmed that M2 TAMs were the primary immune cells expressing LILRB1. Dense infiltration of LILRB1+ M2 TAMs yielded an immunosuppressive microenvironment manifested as enriched exhausted CD8+ T cells and increased immunosuppressive cytokines. Moreover, patients with high infiltration of both LILRB1+ cells and M2 TAMs indicated poor prognosis and inferior therapeutic responsiveness to adjuvant chemotherapy. In conclusion, LILRB1+ M2 TAMs were associated with a pro-tumor immune contexture and determine poor prognosis in gastric cancer. Further studies are essential to explore therapeutic targeting LILRB1+ M2 TAMs.

## Introduction

Gastric cancer (GC) represents the fifth most prevalent malignancy and the third leading cause of cancer mortality worldwide (1). Radical surgery combined with postoperative adjuvant chemotherapy is the main treatment for advanced GC; however, the prognosis of patients is yet poor (2). Due to the uncertainty of chemosensitivity and the consequent chemoresistance, several patients have a high recurrence rate after chemotherapy. The change in the tumor microenvironment can predict the prognosis and chemosensitivity of patients. Therefore, it is crucial to stratify prognosis and predict the treatment response based on tumor microenvironment.

In the tumor microenvironment, tumor-associated macrophages (TAMs) are the primary infiltration components with different forms of expression between inflammation and cancer. Macrophages infiltrating into tumor tissue polarize into an antitumor M1 or a pro-tumor M2 subset. Typically, M2 TAMs exert a specific role in promoting tumor growth, promoting angiogenesis, and inhibiting adaptive immunity. Our previous study also confirmed that M2 TAMs indicate poor prognosis in GC patients (3). Thus, TAMs are an attractive target for tumor therapy. However, increasing evidence showed diversity in macrophages, but distinguishing them only by the current two polarization forms is challenging. Therefore, clarifying the distribution and function of macrophage subsets in tumor tissues is essential for accurate clinical treatment targets.

Leukocyte immunoglobulin-like receptor subfamily B1 (LILRB1) is a transmembrane glycoprotein, a major receptor of human leukocyte antigen G (HLA-G) (4). It is considered to be an immunosuppressive receptor. LILRB1 combines classic and non-classic human major histocompatibility complex (MHC) molecules to exert an immunosuppressive effect. It is mainly involved in the regulation of maternal-fetal immune tolerance and induction of transplantation immune tolerance (5). The rapid growth of tumor cells is largely due to the escape of immune surveillance (6). As an immunosuppressive receptor, LILRB1 may play a major role in the process of tumor cells escaping immune surveillance. Recently, it has been found that macrophages expressing inhibitory receptor LILRB1 interact with MHC class I components on the surface of tumor cells to protect tumor cells from phagocytosis. Moreover, some studies suggested that TAMs affect the efficacy of chemotherapy in tumor patients (7, 8). However, the effect of LILRB1 on TAMs and its role in the tumor microenvironment has not yet been analyzed systematically in GC.

In this study, we detected the frequency of LILRB1 and macrophages in GC patients. The correlation between LILRB1 and clinicopathological factors, macrophage infiltration, and tumor microenvironment immune status would be analyzed. Furthermore, we evaluated the prognostic potential of LILRB1 and macrophages, and assessed the predictive value of postoperative adjuvant chemotherapy in this subpopulation.

## Methods

### Patients and Tissue Samples

The study recruited 166 GC patients who underwent radical surgery during 2009–2013 in the First Affiliated Hospital of Sun Yat-sen University. None of the patients received preoperative treatment, including chemotherapy or radiotherapy. Comprehensive information about the clinicopathological data and survival outcomes of all patients was obtained. The median follow-up period was 42 (range: 2–99) months in this cohort. All tumor tissues and 46 adjacent peritumoral tissues were formalin-fixed and embedded in paraffin. The tumor stages were categorized according to the 7th edition of American Joint Committee on Cancer (AJCC) TNM staging system. Adjuvant chemotherapy (ACT) was given to TNM stage II and III patients after surgery, according to the National Comprehensive Cancer Network (NCCN) guidelines and patient preference. All chemotherapy regimens were fluorouracil-based combination chemotherapy. The human studies were sanctioned by the local ethics committee at the First Affiliated Hospital of Sun Yat-sen University.

### Immunohistochemistry

The formalin-fixed and paraffin-embedded sections were deparaffinized with xylene and then rehydrated. Antigen retrieval was performed with Tris/EDTA buffer pH 9.0 for 20 min at 95 °C in paraffin-embedded tissue sections. The slides were incubated with antibodies against CD163 (1:400; Cell Signaling Technology, #93498) and LILRB1 (1:400; Abcam, ab238145) overnight at 4°C. The reactivity was detected using Dako EnVision-HRP (Dako).

### Assessment of the LILRB1 and CD163 Cell Density in IHC Specimens

The infiltration density of LILRB1+ and CD163+ cells per field was evaluated by two independent pathologists who were blinded to the patients’ clinical data using Image-Pro Plus 6.0 (Media Cybernetics Inc.) for assistance. For each tissue core or normal section, three randomized fields of positive-stained cells were counted under a high-power field (HPF) of 400X. The density of LILRB1+ and CD163+ cells was calculated as the mean number of fields from cores or normal sections. The cut-off values of LILRB1+ and CD163+ cells density were the median values. For LILRB1+ cells,≥85 in average field was defined as high and <85 was defined as low. For CD163+ cells, ≥28 in average field was defined as high and <28 was defined as low.

### Immunofluorescence

We performed immunofluorescence, as described previously. Primary antibodies were used as follows: anti-human CD163 (1:100; Biolegend; 326507), anti-LILRB1 (1:200; Abcam, ab238145), anti-human-Cytokeratin 7 (1:100; Biolegend, 601601). After washing, cells were incubated with Alexa Fluor 488- or 546- or 647-labeled secondary antibodies for 1 h. Nuclei were counterstained using DAPI. The stained cells were visualized using an inverted confocal microscope, and the images were processed using ZEN2.3.

### TCGA and GEO Data Processing

Level 4 gene expression data (RSEM normalized) of The Cancer Genome Atlas (TCGA) were downloaded from the UCSC Xena browser (https://gdc.xenahubs.net). We used TCGA database to analyze the difference of LILRB1 expression between GC and normal tissues. We calculated the scores of LILRB1+ M2 TAMs signature genes by the geomean of TCGA RSEM expression to confirm relative abundance. The correlation between LILRB1+ M2 TAMs and exhausted CD8+ T cells was analyzed by gene set enrichment analysis (GSEA, v3.0), as previously reported (9). The LILRB1+ M2 TAMs signature genes and exhausted CD8+ T cell gene set were showed in **Supplementary Table 1**, which were identified based on previous studies (9, 10).

Microarray datasets GSE15459 and GSE 29272 were downloaded from the Gene Expression Omnibus (GEO) database (http://www.ncbi.nlm.nih.gov/geo/) and used as a training set for the LILRB1 expressed prediction. We also estimated the proportion of immune cells used GSE62254 cohort. The RMA algorithm was applied to normalize and transform all the raw data from GEO to expression values in the R environment (v3.5.3).

### Evaluation of Infiltrating Immune Cells in Public Database

The CIBERSORT algorithm was conducted to evaluate the proportion of immune cells in GC patients, as reported previously (11). This method allows sensitive and specific discrimination of 22 human immune cell phenotypes, including B cells, T cells, natural killer (NK) cells, macrophages, dendritic cells, and myeloid subsets. Briefly, the gene expression profiles were prepared using standard annotation files. Then, the data were uploaded to the CIBERSORT web portal (http://cibersort.stanford.edu/), and the algorithm was run using the default signature matrix at 1000 permutations.

### Statistical Analysis

The correlation between LILRB1 expression and clinicopathological characteristics of GC was evaluated by Student’s t-test. The Pearson’s correlation test was used to determine the extent of correlation between the expression of LILRB1 and that of other genes. The survival outcomes, including overall survival (OS) and disease-free survival (DFS) were analyzed using Kaplan–Meier curves, log-rank test, and univariate/multivariate Cox regression analysis. The data of all groups in the figure were expressed as mean ± SDs. Two-sided P < 0.05 was considered statistically significant. All analyses were performed using GraphPad Prism (version 6.00), R (version 3.6.1) or SPSS statistics (version 21) software.

## Results

### LILRB1 Is Expressed in Stroma of GC and Associated With an Aggressive Phenotype

**Figure 1:**
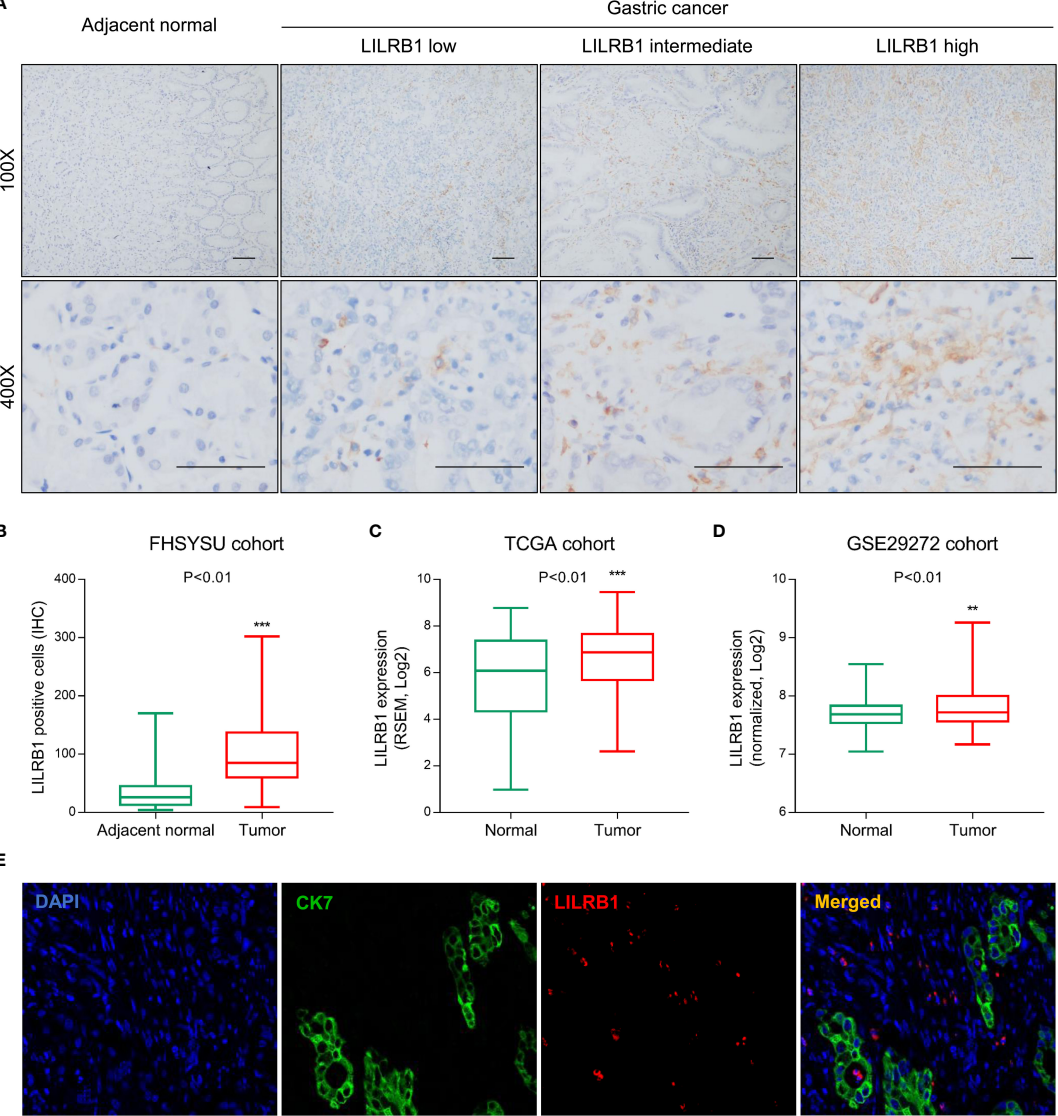
LILRB1 was highly expressed in stroma of GC. **(A)** The expression of LILRB1 in GC tissues and adjacent tissues from First Affiliated Hospital of Sun Yat-sen University (FHSYSU) cohort by IHC. **(B)** Box diagram showed that the count of LILRB1+ cells in GC was higher than that in gastric cancer tissues in FHSYSU cohort. **(C, D)** TCGA cohort and GSE29272 cohort showed higher LILRB1 expression in GC tissues than in normal gastric tissues. **(E)** LILRB1 was mainly distributed in the stroma of gastric cancer by tricolor immunofluorescence microscopy. **P < 0.01 and ***P < 0.001.

Initially, we detected the expression of LILRB1 in GC tissues and adjacent tissues from First Affiliated Hospital of Sun Yat-sen University (FHSYSU) cohort by immunohistochemistry (**Figure 1A**). The current results showed that the expression of LILRB1 was higher in tumor tissues compared to the matched adjacent tissues (P < 0.01, **Figure 1B**). Similarly, we also detected the abnormal expression of LILRB1 in GC tissues in two other independent cohorts (TCGA and GSE29272, **Figures 1C, D**). Interestingly, we found that LILRB1 was mainly present in tumor stroma rather than tumor cells (**Figure 1A**). In order to further determine the expression distribution of LILRB1, immunofluorescence was detected, and GC cells were labeled with cytokeratin 7 (CK7). The data further confirmed that LILRB1 was mainly expressed in the tumor stromal microenvironment (**Figure 1E**).

**Figure 2:**
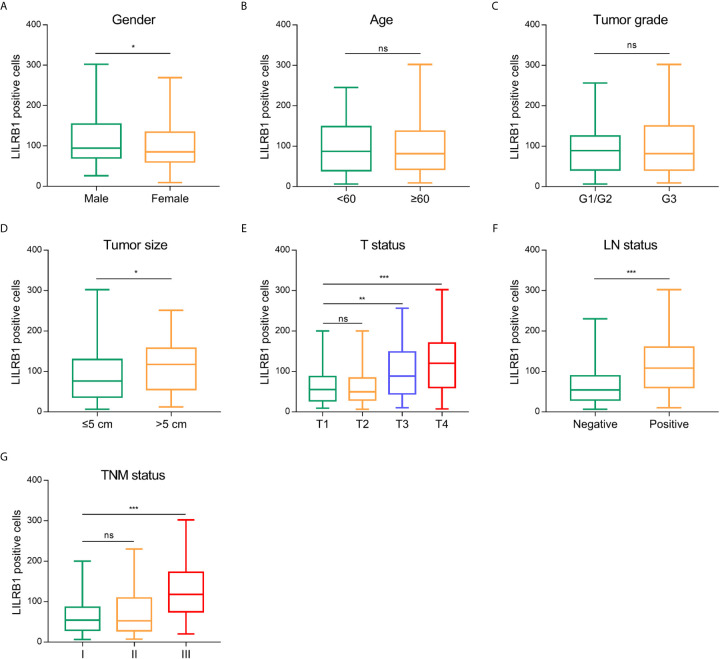
The correlation between LILRB1 and clinicopathological characteristics of GC patients. **(A)** LILRB1 expression in male patients was higher compared with female. **(B, C)** No significant correlation was established between LILRB1 and patients’ age or tumor grade. **(D)** Patients with large tumor size (>5 cm) had high-LILRB1 expression. **(E)** Patients with deep tumor invasion had high-LILRB1 expression. **(F)** Patients with lymph node metastasis had high-LILRB1 expression. **(G)** Higher proportion of LILRB1 was detected in stage III tumors than stage I-II tumors. *P < 0.05, **P < 0.01 and ***P < 0.001. ns, no statistical significance.

Additionally, we analyzed the correlation between LILRB1 and the clinicopathological characteristics of GC patients. We also observed that males with GC expressed more LILRB1 than female patients (**Figure 2A**). However, no significant correlation was established between LILRB1 and patients’ age or tumor grade (**Figures 2B, C**). The LILRB1 expression was significantly associated with large tumor size (>5 cm), deep tumor invasion, and lymph node metastasis (**Figures 2D–F**). Correspondingly, a higher proportion of LILRB1 was detected in stage III tumors than stage I-II tumors (**Figure 2G**).

### M2 TAMs Are the Primary Immune Cells Expressing LILRB1

**Figure 3:**
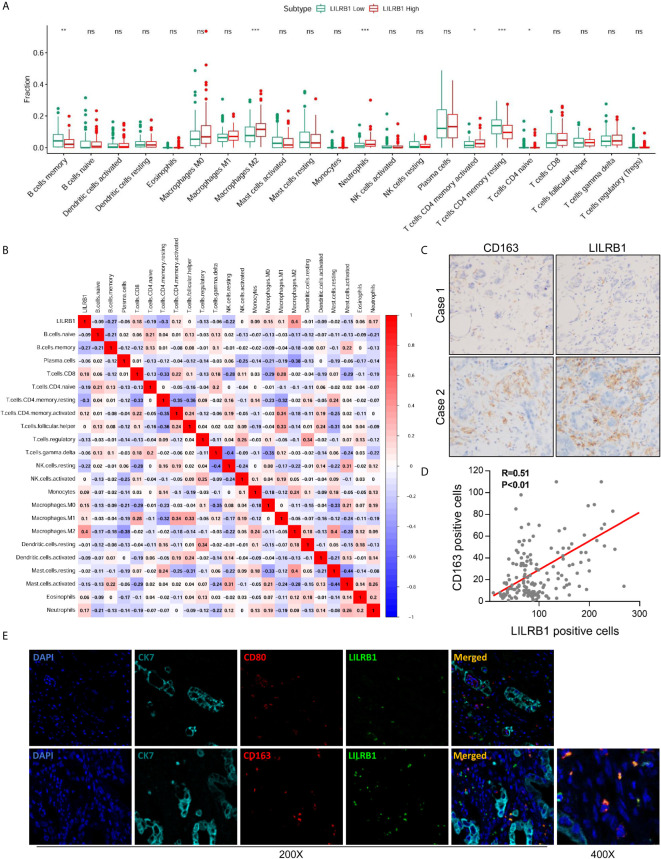
M2 TAMs were the primary immune cells expressing LILRB1. **(A)** CIBERSORT was employed to assess the difference of 22 kinds of immune cells between LILRB1 high tumors and LILRB1 low tumors. **(B)** The correlation analysis between LILRB1 and 22 types of immune cells. **(C, D)** LILRB1 expression showed a positive correlation with M2 macrophages by IHC. **(E)** Immunofluorescence assay showed that LILRB1 was mainly expressed in M2 TAMs. *P < 0.05, **P < 0.01 and ***P < 0.001. ns, no statistical significance.

The tumor microenvironment showed an abundance of immune cell infiltration, which exerted an impact on tumor progression and prognosis of patients (12, 13). Herein, we sought to discover the influence of LILRB1 on the immune contexture in GC. Thus, CIBERSORT was employed to assess the relative proportion of 22 human hematopoietic cell phenotypes (LM22) within the GSE15459 database. The data showed that high LILRB1 expression was associated with a low level of memory B cells and memory resting CD4 T cells but a high proportion of M2 TAMs, neutrophils, and memory-activated CD4 T cells (**Figure 3A**). To further analyze the correlation between LILRB1 and the immune microenvironment of GC, we conducted a correlation analysis between LILRB1 and 22 types of immune cells. Next, we found an interesting phenomenon that LILRB1 had a significant positive correlation with M2 TAMs infiltration (**Figure 3B**), considered immunosuppressive cells that promote tumor progression (14). However, no significant correlation was established between LILRB1 and M1 macrophage infiltration, which was previously identified to possess the antitumor effect in GC (3).

To substantiate these findings, we performed IHC staining of LILRB1 and CD163 (classic biomarker of M2 TAMs). Consistent with the results from CIBERSORT, LILRB1 expression showed a positive correlation with M2 TAMs (**Figures 3C, D**, Pearson’s correlation R = 0.51, P < 0.01). Subsequently, we conducted immunofluorescence staining to evaluate the correlation between LILRB1 expression and macrophage localization. As illustrated in **Figure 3E**, M2 TAMs were identified as components of the LILRB1 infiltrate, expressing the CD163 M2 marker. In contrast, no co-expression of LILRB1 and CD80 (biomarker of M1 macrophages) was detected.

### LILRB1 Expression Is Correlated With Multiple M2 Macrophage-Related Markers

**Figure 4:**
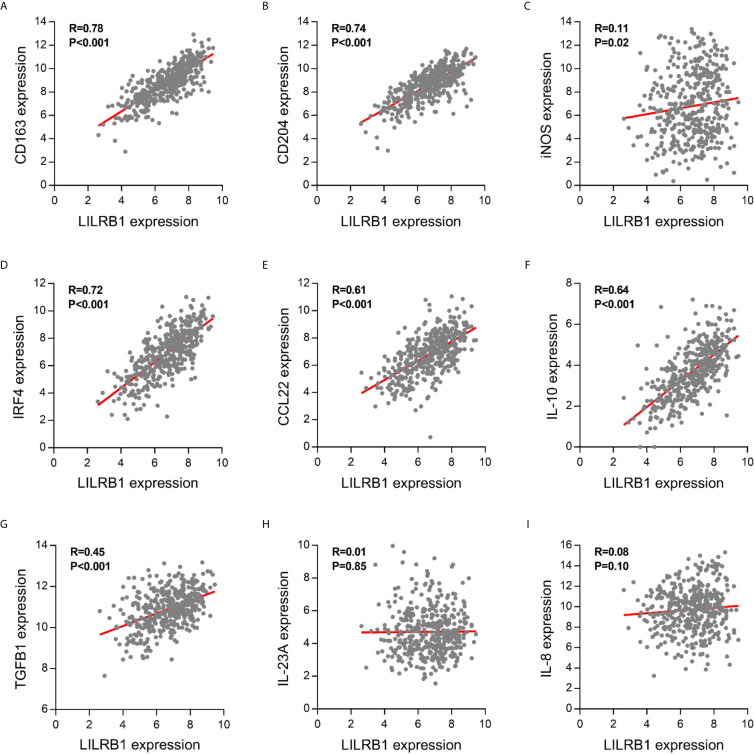
LILRB1 expression was correlated with multiple M2 macrophage-related molecules. Correlation between LILRB1 and M1/M2 macrophage-related molecules including **(A)** CD163, **(B)** CD204, **(C)** iNOS, **(D)** IRF4, **(E)** CCL22, **(F)** IL-10, **(G)** TGF-β1, **(H)** IL-23A, and **(I)** IL-8.

Further, we analyzed the macrophage markers in the TCGA database to verify the CIBERSORT results. These findings showed that LILRB1 expression was positively correlated with the expression of CD163 and CD204, M2 macrophage markers, involved in promoting tumor growth and metastasis (**Figures 4A, B**). On the other hand, no significant correlation was observed between LILRB1 and iNOS expression, which was widely recognized as a marker of M1 macrophages (**Figure 4C**). Moreover, transcription factor IRF4 induced M2-type polarization of macrophages, which was upregulated in patients with high LILRB1 expression (**Figure 4D**) (15).

The imbalance between proinflammatory and anti-inflammatory cytokines secreted by M1/M2 macrophages in the tumor microenvironment promote the development of GC (16). Therefore, we hypothesized that cytokines are dysregulated in high-LILRB1 expression tumors. M2 macrophage-derived cytokines promote an immunosuppressive tumor microenvironment, including CCL22, IL-10, and TGF-β1, that were significantly correlated with LILRB1 expression GC patients (**Figures 4E–G**). Conversely, M1 macrophage-derived proinflammatory cytokines, such as IL-23A and IL-8, were not correlated with LILRB1 expression (**Figures 4H, I**). Thus, these results suggested that LILRB1 may be involved in the M2 polarization of macrophages to promote GC progression.

### LILRB1+ M2 TAMs Exhibit an Immunosuppressive Phenotype

**Figure 5:**
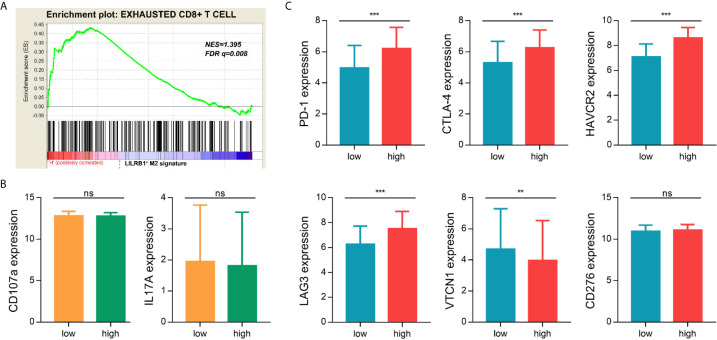
LILRB1+ M2 TAMs exhibited an immunosuppressive phenotype. **(A)** GSEA suggested that the exhausted CD8+ T cells genes enriched in LILRB1+ M2 TAMs signature high GC patients. **(B)** Expression difference of effector molecules (CD107a, IL-17A) between LILRB1+ M2 TAMs high signature and low signature. **(C)** Expression of immune checkpoint molecules (PD-1, CTLA-4, HAVCR2, LAG-3, VTCN1, and CD276) in LILRB1+ M2 TAMs high and low expression subgroup. **P < 0.01 and ***P < 0.001, ns, no statistical significance.

We next aimed to investigate the potential impact of LILRB1-expressing M2 TAMs on immune microenvironment in GC. We conducted GSEA to analyze the relationship between LILRB1+ M2 TAMs and functional status of CD8+ T cells. The result showed that exhausted CD8+ T cell gene set was significantly enriched in high LILRB1+ M2 TAMs signature (**Figure 5A**, FDR q = 0.008). There was no significant difference in the expression of effector molecules including CD107a and IL-17A in classification of LILRB1+ M2 TAMs signature (**Figure 5B**). Moreover, we evaluated the relationship between LILRB1+ M2 TAMs and immune checkpoint molecules. High LILRB1+ M2 TAMs signature exhibited abundant programmed cell death protein 1 (PD-1), cytotoxic T-lymphocyte associated protein 4 (CTLA-4), lymphocyte activation gene-3 (LAG-3), and hepatitis A virus cellular receptor 2 (HAVCR2) expression (**Figure 5C**). Consequently, these data indicated that LILRB1+ M2 TAMs may be involved in promoting immune escape of GC cells.

### LILRB1 and M2 TAMs Determine Poor Prognosis in GC Patients

**Figure 6:**
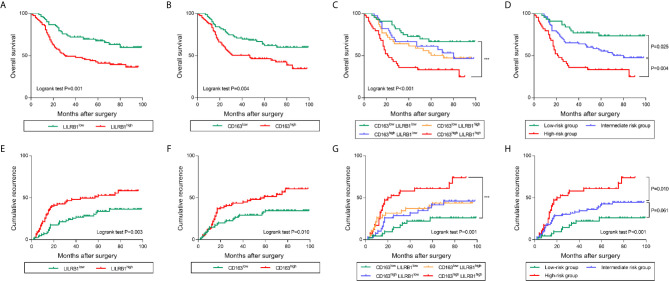
LILRB1 and M2 TAMs determined poor prognosis in GC patients. **(A, B)** Overall survival curves according to the levels of LILRB1 and CD163 distribution in GC patients. **(C, D)** Overall survival of GC patients stratified on the basis of LILRB1 and CD163. **(E, F)** Recurrence curve according to the levels of LILRB1 and CD163 distribution. **(G, H)** Recurrence risk stratified on the basis of LILRB1 and CD163. ***P < 0.001.

To further discover the clinical significance of LILRB1 and M2 TAMs infiltration in GC, we evaluated the prognosis of LILRB1+ and CD163+ cells by Kaplan–Meier analysis and log-rank test. The findings revealed that GC patients with high expression of LILRB1 had a poor OS (**Figure 6A**). High density of CD163+ macrophage infiltration also predicted unfavorable prognosis in OS (**Figure 6B**). Moreover, high levels of LILRB1 and CD163 were associated with a high risk of recurrence in GC patients (**Figures 6E, F**). Multivariate Cox regression analysis demonstrated that differentiation grade (OS: hazard ratio (HR): 2.025, 95% CI: 1.148-3.571, P = 0.015; DFS: HR: 3.083, 95% CI: 1.569-6.059, P = 0.001), high CD163 (OS: HR 1.866, 95% CI: 1.177-2.958, P = 0.008; DFS: HR: 1.771, 95% CI: 1.073-2.925, P = 0.025), high LILRB1 expression (OS: HR: 2.008, 95% CI: 1.262-3.195, P = 0.003; DFS: HR: 1.947, 95% CI: 1.187-3.195, P = 0.008), and TNM stage (OS: HR: 3.095, 95% CI: 1.465-6.540, P = 0.003; DFS: HR: 3.702, 95% CI: 1.619-8.464, P = 0.002) was an independent poor prognostic factor for OS and DFS (**Tables 1**, **2**). We also evaluated the correlation between the expressions of LILRB1 and CD163 and OS by every stage. There was a significant positive correlation between LILRB1 and CD163 expression in different TNM stages. Patients with high expression of LILRB1 had poorer OS in TNM stage II and III. However, the relationship between LILRB1 and OS in TNM stage I patients was not statistically significant, possibly due to the small number of stage 1 patients included in the study. These findings were showed in **Supplementary Figure 1**.

**Table 1:** Univariate and multivariate analyses for OS in GC patients.

Variable	Univariate			Multivariate		
	HR	95% CI	*P*	HR	95% CI	*P*
Age (years)						
≥60 *vs*. <60	1.188	0.764-1.847	0.443			
Sex						
Male *vs*. female	1.449	0.897-2.343	0.130			
Grade						
G3 *vs*. G1/G2	1.770	1.032-3.035	0.038	2.025	1.148-3.571	0.015
Tumor size						
>5 cm *vs*. ≤5 cm	1.451	0.907-2.321	0.120			
Tumor depth						
T3-4 *vs*. T1-2	2.180	1.288-3.690	0.004	1.400	0.688-2.848	0.253
Lymph node involvement						
Present *vs*. none	1.886	1.184-3.005	0.008	0.731	0.351-1.524	0.404
CD163						
High *vs*. low	1.914	1.217-3.010	0.005	1.866	1.177-2.958	0.008
LILRB1						
High *vs*. low	2.158	1.365-3.413	0.001	2.008	1.262-3.195	0.003
TNM stage						
III *vs*. I-II	2.960	1.874-4.675	<0.001	3.095	1.465-6.540	0.003

**Table 2:** Univariate and multivariate analyses for DFS in GC patients.

Variable	Univariate			Multivariate		
	HR	95% CI	*P*	HR	95% CI	*P*
Age (years)						
≥60 *vs*. <60	0.947	0.587-1.530	0.825			
Sex						
Male *vs*. female	1.442	0.861-2.415	0.164			
Grade						
G3 *vs*. G1/G2	2.592	1.356-4.951	0.004	3.083	1.569-6.059	0.001
Tumor size						
>5 cm *vs*. ≤5 cm	1.290	0.771-2.159	0.332			
Tumor depth						
T3-4 *vs*. T1-2	2.851	1.556-5.223	0.001	1.303	0.603-2.817	0.501
Lymph node involvement						
Present *vs*. none	2.091	1.249-3.449	0.005	0.717	0.315-1.628	0.426
CD163						
High *vs*. low	1.885	1.154-3.078	0.011	1.771	1.073-2.925	0.025
LILRB1						
High *vs*. low	2.048	1.225-3.343	0.004	1.947	1.187-3.195	0.008
TNM stage						
III *vs*. I-II	3.256	1.983-5.346	<0.001	3.702	1.619-8.464	0.002

Furthermore, we combined LILRB1+ cells with CD163+ cells for survival analysis. Notably, patients with high infiltration of both LILRB1+ and CD163+ cells indicated poor OS and high risk of recurrence (**Figures 6C, G**). Low levels of LILRB1+ and CD163+ cells infiltration predicted favorable survival. Patients with LILRB1highCD163low or LILRB1lowCD163high levels infiltration showed an intermediate prognosis. In order to highlight the prognostic merit and enhance clinical practicality, we trichotomized patients into three risk subgroups: low-risk group (LILRB1lowCD163low), the intermediate-risk group (LILRB1highCD163low/LILRB1lowCD163high), and high-risk group (LILRB1highCD163high). Consistent with our identification, the low-risk group showed a favorable prognosis, while high-risk group showed poor OS and maximal risk of recurrence (**Figures 6D, H**).

### Increased LILRB1+ TAM Infiltration Predicts Poor Efficacy of Adjuvant Chemotherapy After Surgery in GC Patients

**Figure 7:**
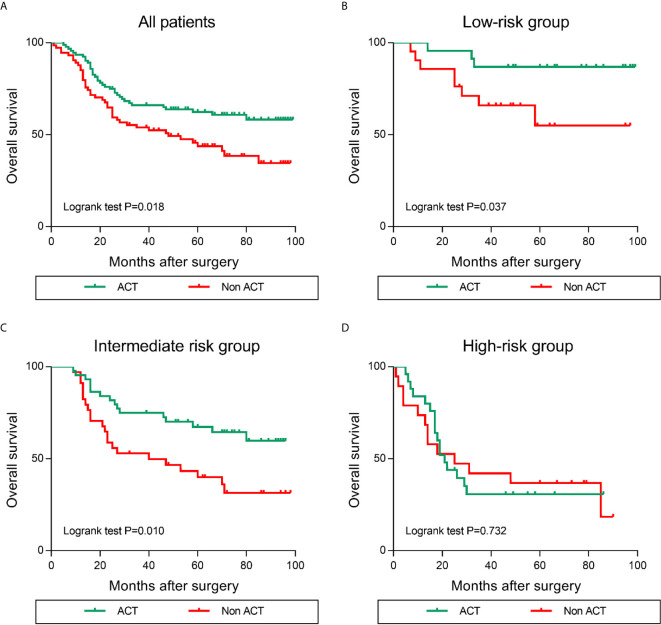
Increased LILRB1 and M2 TAMs infiltration predict poor efficacy of ACT (adjuvant chemotherapy) in GC patients. Overall survival for **(A)** all enrolled, **(B)** low-risk, **(C)** intermediate-risk, **(D)** high-risk GC patients with or without ACT.

Previous studies demonstrated that the alternations within tumor infiltrating immune contexture predominantly affected the response to postoperative adjuvant chemotherapy (ACT) (17). Thus, we assessed the predictive value of different risk groups for the efficacy of fluorouracil-based ACT in GC patients. Typically, ACT produced survival benefits in the GC patients (**Figure 7A**). The results of stratified analysis suggested that patients in the low and intermediate risk group benefit significantly from ACT (**Figures 7B, C**). However, in patients with high risk (LILRB1highCD163high), OS was not improved even after ACT was applied (**Figure 7D**). Taken together, these findings revealed that the levels of LILRB1+ cells combined with CD163+ cells could stratify patients into various risk subgroups and predict the sensitivity of patients to ACT.

## Discussion

Leukocyte immunoglobulin-like receptor subfamily B (LILRB) is a transmembrane glycoprotein and is a critical receptor of human leukocyte antigen G (HLA-G) molecules (18). It is widely distributed and expressed in NK cells, monocytes/macrophages, dendritic cells (DCs), and tumor cells (19). Previous studies have found that LILRB1 promotes tumor development, such as lung cancer, breast cancer, and pancreatic cancer, which can significantly enhance the movement and migration ability of cancer cells and promote tumor metastasis. Furthermore, LILRB1 is considered as an immunosuppressive receptor that can not only bind with the classical human major histocompatibility complex (MHC) molecules but also with non-classical MHC molecules (such as HLA-G and HLA-E) to exert immunosuppressive effect (20). It is mainly involved in the regulation of maternal fetal immune tolerance and induction of transplantation immune tolerance. The rapid growth of tumor cells could be attributed to the escape of immune surveillance. As a major immunosuppressive receptor, LILRB1 plays a critical role in the escaping of tumor cells from immune surveillance. Thus, it is of great significance to understand the function of LILRB1 and break the “tumor immune tolerance microenvironment.”

In recent years, some studies have shown that LILRB1 is expressed in a variety of tumor cells, and the expression level is significantly related to tumor growth and prognosis of patients (21). For example, LILRB1 promotes tumor progression in maintaining the stem cells and hematopoietic stem cells (22). Interestingly, LILRB1 is mainly expressed around tumor cells rather than on tumor cells in patients with GC. The analysis of GSE15459 cohort and immunohistochemical detection of 166 patients with GC in our center revealed that the expression of LILRB1 was positively correlated with M2 TAMs infiltration. Immunofluorescence further confirmed that M2 macrophages are the main immune cells expressing LILRB1, rather than M1 macrophages. M2 TAMs are the most abundant immune cells in tumor tissues. M2 TAMs stimulate natural T cells to produce Th2 type response. These cells secrete vascular endothelial growth factor (VEGF), transforming growth factor β (TGF-β), and other cytokines (23) that promote tissue repair, angiogenesis, immunosuppression, and tumor progression (24). It has been found that LILRB1 high-expressing macrophages interact with MHC class I components on tumor cell surfaces to protect tumor cells from phagocytosis (25). In addition, LILRB1 promotes M2 polarization of macrophages. These results suggested that LILRB1 mediates M2 TAMs to promote tumor immune escape in GC, which is a potential target for antitumor immunotherapy.

We further investigated the impact of LILRB1 on immune microenvironment in GC patients. LILRB1 expression was significantly correlated with several M2 macrophage-related cytokines including CCL22, IL-10, and TGF-β1. These molecules have been widely reported to promote tumor metastasis, immune escape, and angiogenesis (26–29). We next explored the difference of immune status between LILRB1+ M2 TAMs high and low subgroups. The exhausted CD8+ T cell gene set was found significantly enriched in LILRB1+ M2 TAMs signature high GC patients. Exhaustive T cells are a group of T cells with reduced effector function and continued expression of inhibitory receptors (30). It is involved in the negative regulation of tumor immunity (31). Additionally, high LILRB1+ M2 TAMs signature exhibited abundant immune checkpoint molecules expression including PD-1, CTLA-4, LAG-3, and HAVCR2. These results suggest that the application of corresponding monoclonal antibodies targeting immune regulatory points to reverse depleted T cells and restore anti-tumor immune response may benefit GC patients with high LILRB1 expression.

Prognosis evaluation is the key to selecting the appropriate treatment for cancer patients. In recent years, the prognostic significance of tumor-infiltrating immune cells has gained increasing attention because of their role in the occurrence and development of tumors (32). In the current study, we confirmed that patients with LILRB1 and CD163+ cell infiltration had poor OS rate and high recurrence rate. Moreover, we divided three risk subgroups according to the expression levels of LILRB1 and CD163. Patients with increased expression of LILRB1 and CD163 show poor prognosis. Adjuvant chemotherapy has been recommended as the standard therapy to improve the prognosis of patients with stage II/III GC. However, all patients do not benefit from adjuvant chemotherapy, and the selection criteria of candidate regimens are yet unclear. Recent studies suggested that tumor-associated macrophages affect the efficacy of chemotherapy in tumor patients (8). Therefore, we further studied the correlation between the infiltration of LILRB1 and CD163+cells and the efficacy of chemotherapy in patients with stage II/III GC. Notably, when a large number of LILRB1 and CD163+ cells infiltrate into GC tissues, patients may not benefit from chemotherapy. These results would facilitate appropriate selection of adjuvant chemotherapy for the management of GC patients.

Nevertheless, the present study has serval limitations. First, our study is based on a retrospective design. An external cohort is required to verify the prognostic significance of LILRB1 in GC patients and ACT efficacy. Moreover, there is no international unification cutoff value to identify the levels of LILRB1 and CD163 expression. Different cutoff values may affect the repeatability of the results. Moreover, we have not identified the mechanism underlying the formed and differentiated LILRB1+ macrophages, which need to be explored in future research. Previous studies have reported that the numbers of CD163+ macrophages were higher in tumor microenvironment of cases with a cytotoxic/Th1 signature (33, 34). Thus, using CD163 alone as a marker of M2 macrophages is not rigorous enough. To supplement this deficiency, we also found a correlation between LILRB1 and M2 macrophage expression using CIBERSORT algorithm. In different cohort, our analysis also found that LILRB1 was associated with the expression of other M2 macrophage marker (CD204).

## Conclusions

This study revealed that LILRB1 is highly expressed in GC tissues and mainly expressed in M2 macrophages. Dense infiltration of LILRB1+ M2 TAMs yielded an immunosuppressive microenvironment. Patients with high infiltration of both LILRB1+ cells and M2 TAMs indicated poor prognosis and inferior therapeutic responsiveness to adjuvant chemotherapy. Further studies are essential to explore therapeutic targeting LILRB1+ M2 TAMs.

## Data Availability Statement

The original contributions presented in the study are included in the article/**Supplementary Material**. Further inquiries can be directed to the corresponding authors.

## Ethics Statement

The studies involving human participants were reviewed and approved by the institutional review board and ethics committee of the Seventh Affiliated Hospital of Sun Yat-sen University, Shenzhen, Guangdong, China. The patients/participants provided their written informed consent to participate in this study.

## Author Contributions

SY, YH, and CZ conceived and designed the study. SY, YZ, and HW performed experiments and analyzed the data. YZ, HW, XX, and HL wrote the paper. SY, TH, YH, and CZ edited the manuscript and provided critical comments. All authors contributed to the article and approved the submitted version.

## Funding

This study was funded by grants from National Natural Science Foundation of China (82003104, 82073148, and 81772579), Guangdong Provincial Key Laboratory of Digestive Cancer Research (2021B1212040006), Guangdong Basic and Applied Basic Research Foundation (2019A1515110632), Sanming Project of Medicine in Shenzhen (SZSM201612022), Shenzhen Sustainable Project (KCXFZ202002011010593), China Postdoctoral Science Foundation (2021T140768) and Postdoctoral Foundation of the Seventh Affiliated Hospital of Sun Yat-sen University (ZSQYRSFPD0003). All these study sponsors have no roles in the study design, in the collection, analysis and interpretation of data.

## Conflict of Interest

The authors declare that the research was conducted in the absence of any commercial or financial relationships that could be construed as a potential conflict of interest.

## Publisher’s Note

All claims expressed in this article are solely those of the authors and do not necessarily represent those of their affiliated organizations, or those of the publisher, the editors and the reviewers. Any product that may be evaluated in this article, or claim that may be made by its manufacturer, is not guaranteed or endorsed by the publisher.

## Supplementary Material

Supplementary Figure 1 Correlation analysis of LILRB1 with CD163 expression and OS was stratified according to TNM stage. **(A)** Correlation between LILRB1 and CD163 expression in TNM stage I, II and III. **(B)** Correlation between LILRB1 and OS in TNM stage I, II and III.
