# Supplementary figures and images for: Poor Prognosis and Therapeutic Responses in LILRB1-Expressing M2 Macrophages-Enriched Gastric Cancer Patients
Source: Front Oncol. 2021 Aug 9;11:668707. doi: 10.3389/fonc.2021.668707 (PMC8415088; doi:10.3389/fonc.2021.668707)

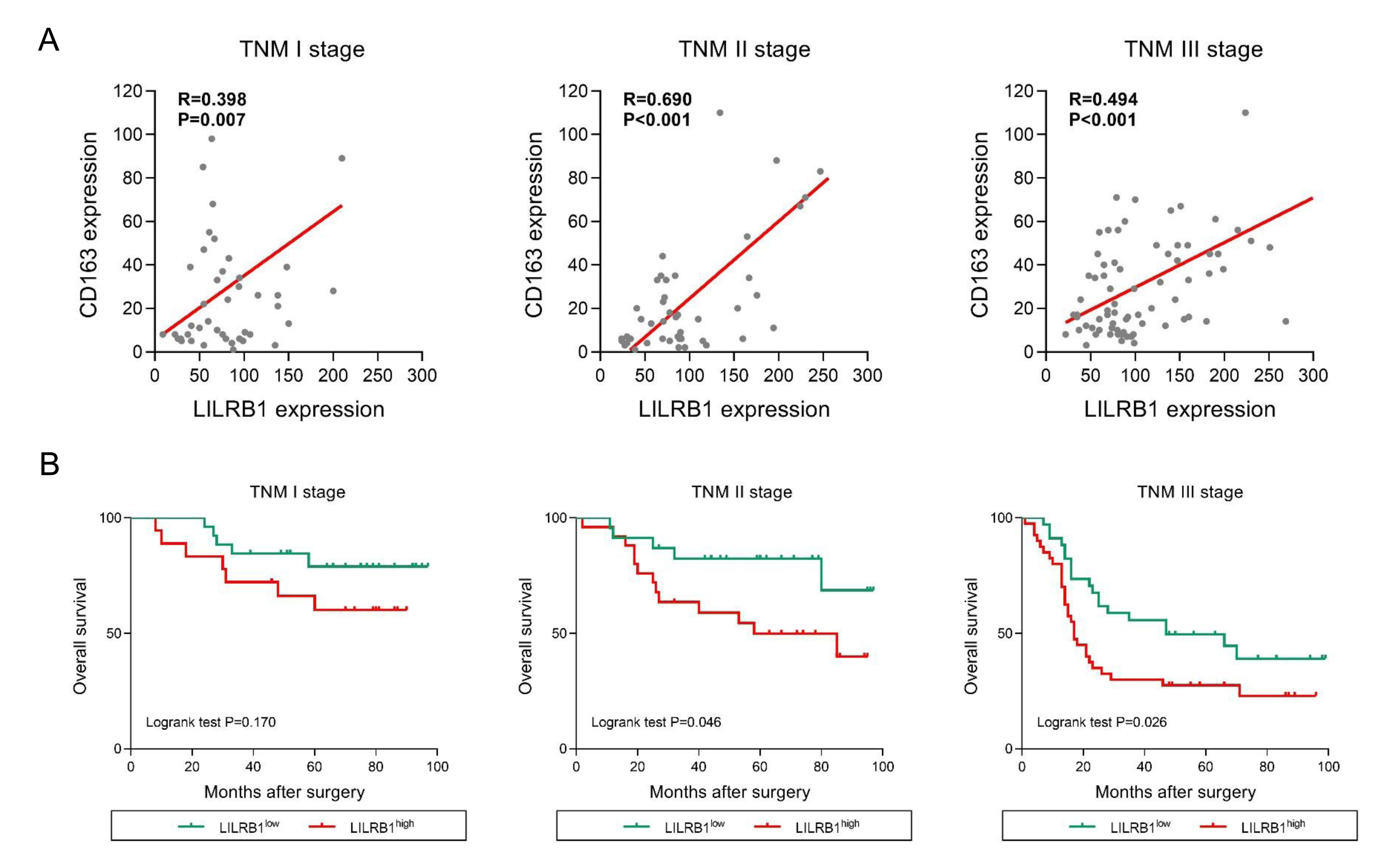

Supplement: Supplementary Figure 1 — Correlation analysis of LILRB1 with CD163 expression and OS was stratified according to TNM stage. (A) Correlation between LILRB1 and CD163 expression in TNM stage I, II and III. (B) Correlation between LILRB1 and OS in TNM stage I, II and III. [file Image_1.jpeg]
