# Supplementary material for: Poor Prognosis and Therapeutic Responses in LILRB1-Expressing M2 Macrophages-Enriched Gastric Cancer Patients
Source: Front Oncol. 2021 Aug 9;11:668707. doi: 10.3389/fonc.2021.668707 (PMC8415088; doi:10.3389/fonc.2021.668707)
Supplement: Supplementary file 2 [file Table_1.docx]

Supplementary Material

# Supplementary Table

Table S1. The LILRB1^+^ M2 TAMs signature genes and exhausted CD8+ T cell gene set

| LILRB1+ M2 TAMs signature genes | LILRB1, CCL14, CCL18, CCL23, CD4, CD68, CLEC4A, CRYBB1, FRMD4A, HRH1, MS4A6A, TXNDC3, NPL, RENBP, WNT5B |
| --- | --- |
| exhausted CD8+ T cell genes | ABCG1, ACADVL, ACSL1, ADAM7, AFP, AGAP1, AHR, ANXA3, APP, ATP2A2, ATP5J2, AUH, BET1, C10orf58, C12orf41, C16orf72, C17orf79, C3orf78, C8orf85, C9orf114, C9orf3, CADM1, CANX, CARM1, CCRL2, CD22, CD244, CELA1, CELF4, CFH, CFHR2, CHL1, CKMT2, CLCA1, CLDN11, COCH, COL19A1, CPA3, CPSF2, CRISP2, CSF1, CXCL13, CXCL14, CXorf26, CYP2A6, CYP4V2, DDIT4, DFFA, DOCK7, DPP7, DUSP6, EFNB3, EFS, EGR2, EIF2AK2, ENPP2, EOMES, EPCAM, ERCC5, EVI5, EXOSC8, F2RL1, FAM101B, FAM207A, FGF6, FHL1, FRK, GABRR2, GATA2, GCET2, GCM2, GDAP1, GDNF, GMCL1, GNAO1, GPLD1, GPM6B, GPR56, GSTM3, GSTO1, GTF3C4, H19, HAO2, HINFP, HIST1H1E, HIST1H3F, HLA-DMA, HMGA2, HOXC6, HTRA2, IFIH1, IGF1R, IL1A, IMMT, INCA1, IRF6, IRS1, KCNAB1, KCTD12, KIAA1217, LCLAT1, LHCGR, LIN9, LOC81691, MAGEL2, MAP1S, MAP2, MCAM, MDN1, METAP2, MITF, MRPL48, MRPS2, MSX1, MTRF1L, MYO6, NAP1L2, NCAN, NDUFA13, NEFH, NEUROD1, NFIB, NKIRAS1, NKX2-2, NOTCH4, NQO1, NR4A2, NRK, NSDHL, OVGP1, P2RX4, PAWR, PAX1, PCLO, PER2, PHLDA3, PHLDB2, PIK3C2G, PLA2G10, PLEKHA1, PNRC1, POLR1B, POLR2C, POU2F1, PTGER2, PTK6, PTPN12, PTPRJ, RBM15, RDBP, RGS10, RGS16, RHAG, RIN2, RPRD1B, SCAMP1, SCAND1, SCN1A, SCN7A, SCRG1, SIX1, SLC12A2, SLC30A1, SLC6A4, SLC7A11, SMAD1, SPOCK2, SPP1, SPRED2, SSBP2, STRA6, SUB1, SYT1, TAPBP, TBX15, TCF4, TERF1, TGM2, TLR7, TM2D1, TM2D3, TM4SF1, TMEM150A, TMEM5, TNFRSF4, TNFRSF9, TRPC1, TUG1, TWSG1, VAMP7, VCAM1, WFS1, WLS, YAP1, ZFP28, ZNF239, ZNF35, ZNF821, ZNRF1, ZRANB1 |
